# Supplementary material for: Significantly wetter or drier future conditions for one to two thirds of the world’s population
Source: Nat Commun. 2024 Jan 11;15:483. doi: 10.1038/s41467-023-44513-3 (PMC10784476; doi:10.1038/s41467-023-44513-3)
Supplement: Supplementary file 1 — Supplementary Information [file 41467_2023_44513_MOESM1_ESM.pdf]

# Supplementary Information for

# Significantly wetter or drier future conditions for one to two thirds of the world's population

Ralph Trancoso[1,2], Jozef Syktus[1], Richard P Allan[3], Jacky Croke[4], Ove Hoegh-Guldberg[1], Robin Chadwick[5,6]

1. School of The Environment, The University of Queensland, Australia
2. Climate Projections and Services, Department of Environment and Science, Queensland Government, Australia
3. Department of Meteorology and National Centre for Earth Observation, University of Reading, United Kingdom
4. Centre for Climate, Environment and Sustainability, School of Earth and Atmospheric Sciences, Queensland University of Technology, Australia
5. Met Office Hadley Centre, Exeter, United Kingdom
6. Global Systems Institute, Department of Mathematics, University of Exeter, United Kingdom

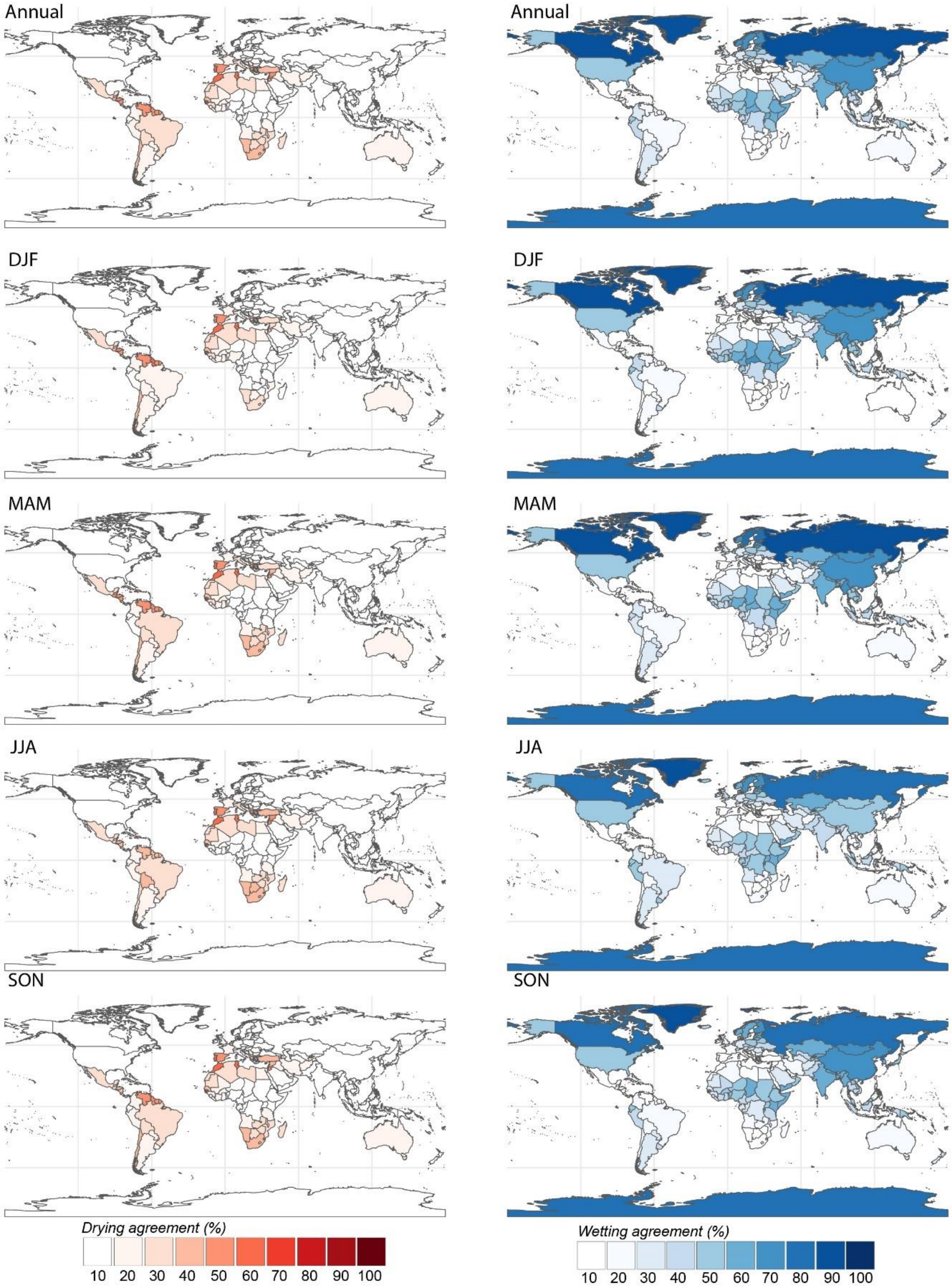


**Figure S1.** Multi-model drying and wetting agreement for 67 GCMs (25 CMIP5 and 42 CMIP6 models) under intermediate emissions (RCP4.5 /SSP2-4.5) for annual and calendar seasons across countries (see Supplementary Data 1 for additional country details and Supplementary Data 2 for state-level analysis).

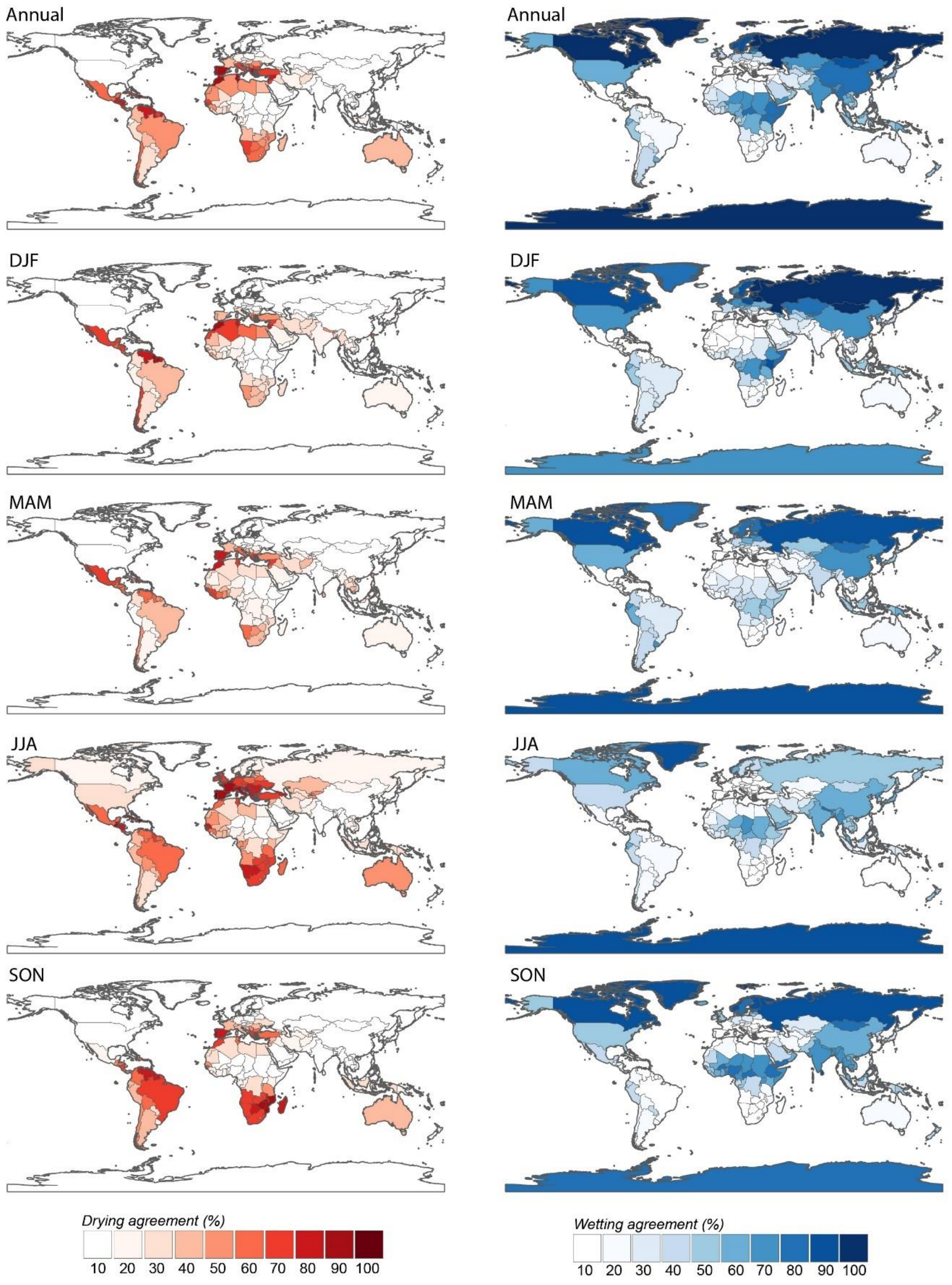


**Figure S2.** Multi-model drying and wetting agreement for 79 GCMs (35 CMIP5 and 44 CMIP6 models) under very high emissions (RCP8.5 /SSP5-8.5) for annual and calendar seasons across countries (see Supplementary Data 1 for additional country details and Supplementary Data 2 for state-level analysis).

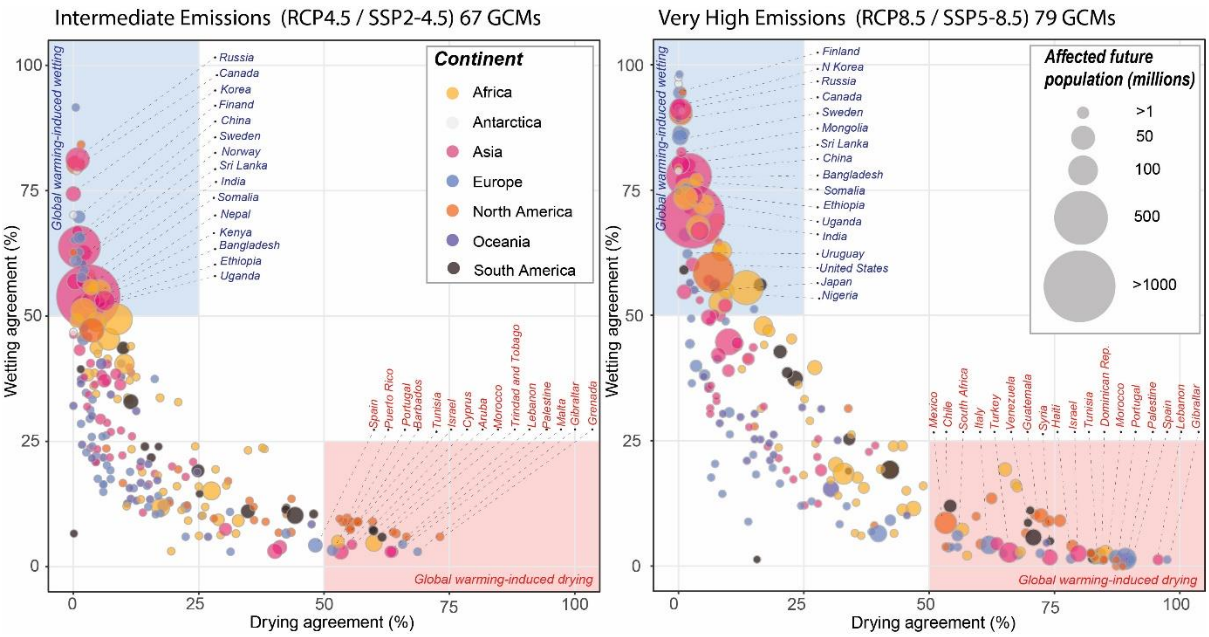


**Figure S3.** Future population affected by global warming-induced wetting and drying based on the agreement of long-term annual precipitation trends of 146 CMIP5 and CMIP6 GCMs forced under intermediate and very high emissions. Country-level regionalization of drying and wetting agreement and future affected population. Colours denote continents and bubble size shows population. Red and blue rectangles indicate the regions where the majority of GCMs agree. Countries with higher agreement, population and representative of different regions are labelled.

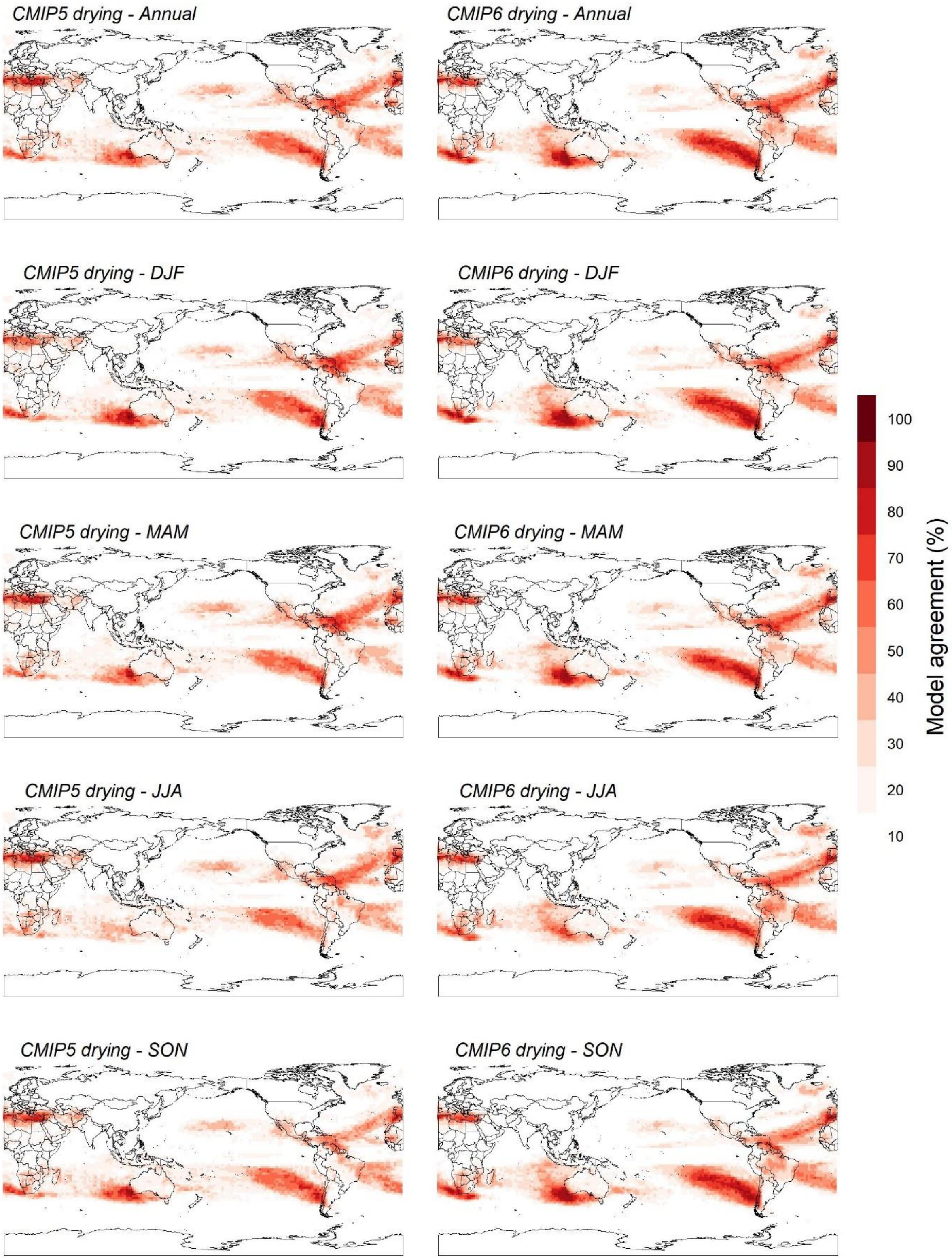


**Figure S4.** Multi-model drying agreement for 25 CMIP5 and 42 CMIP6 models for annual and calendar seasons obtained by long-term non-parametric trends under intermediate emissions (RCP4.5 / SSP2-4.5).

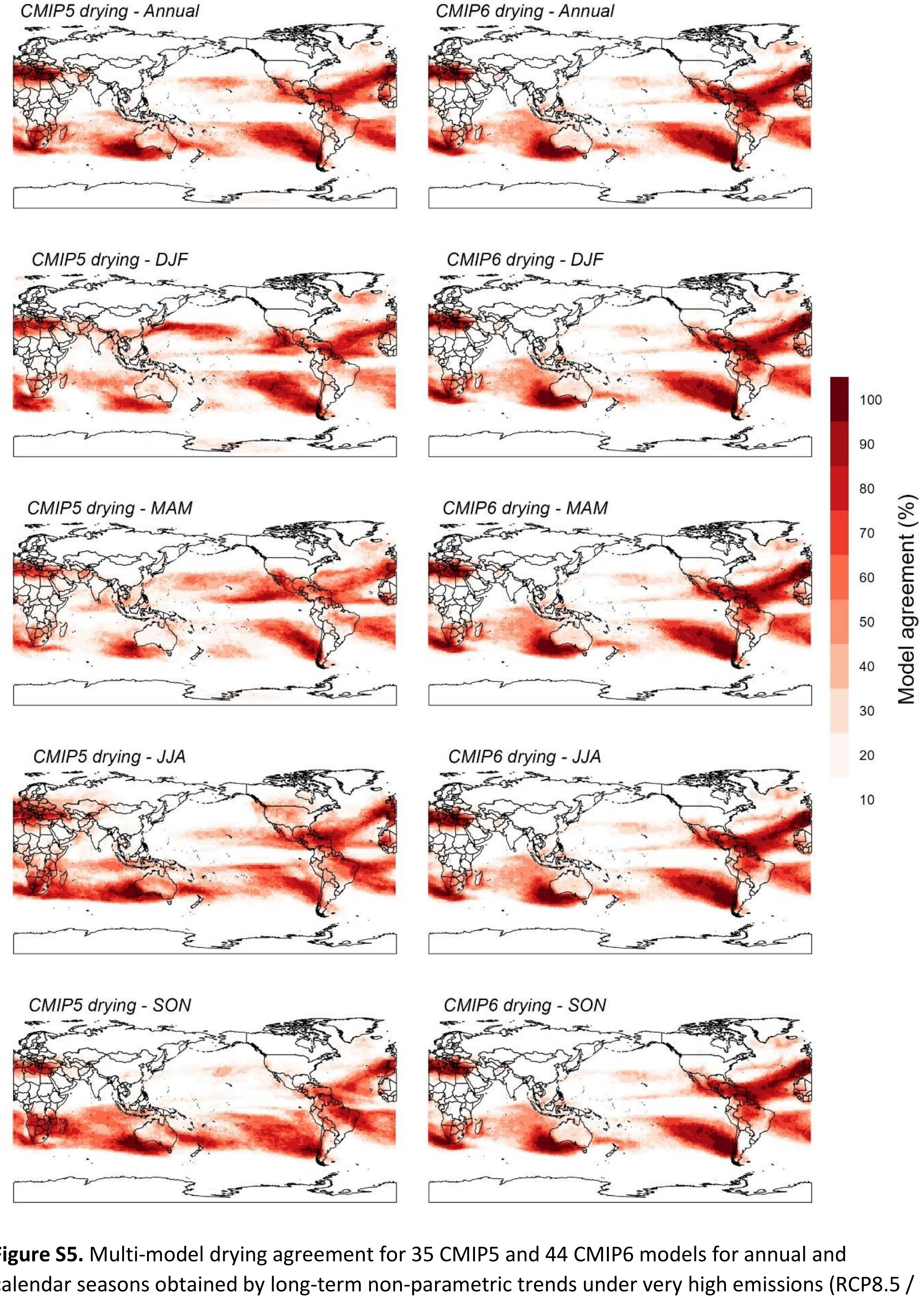


**Figure S5.** Multi-model drying agreement for 35 CMIP5 and 44 CMIP6 models for annual and calendar seasons obtained by long-term non-parametric trends under very high emissions (RCP8.5 / SSP5-8.5).

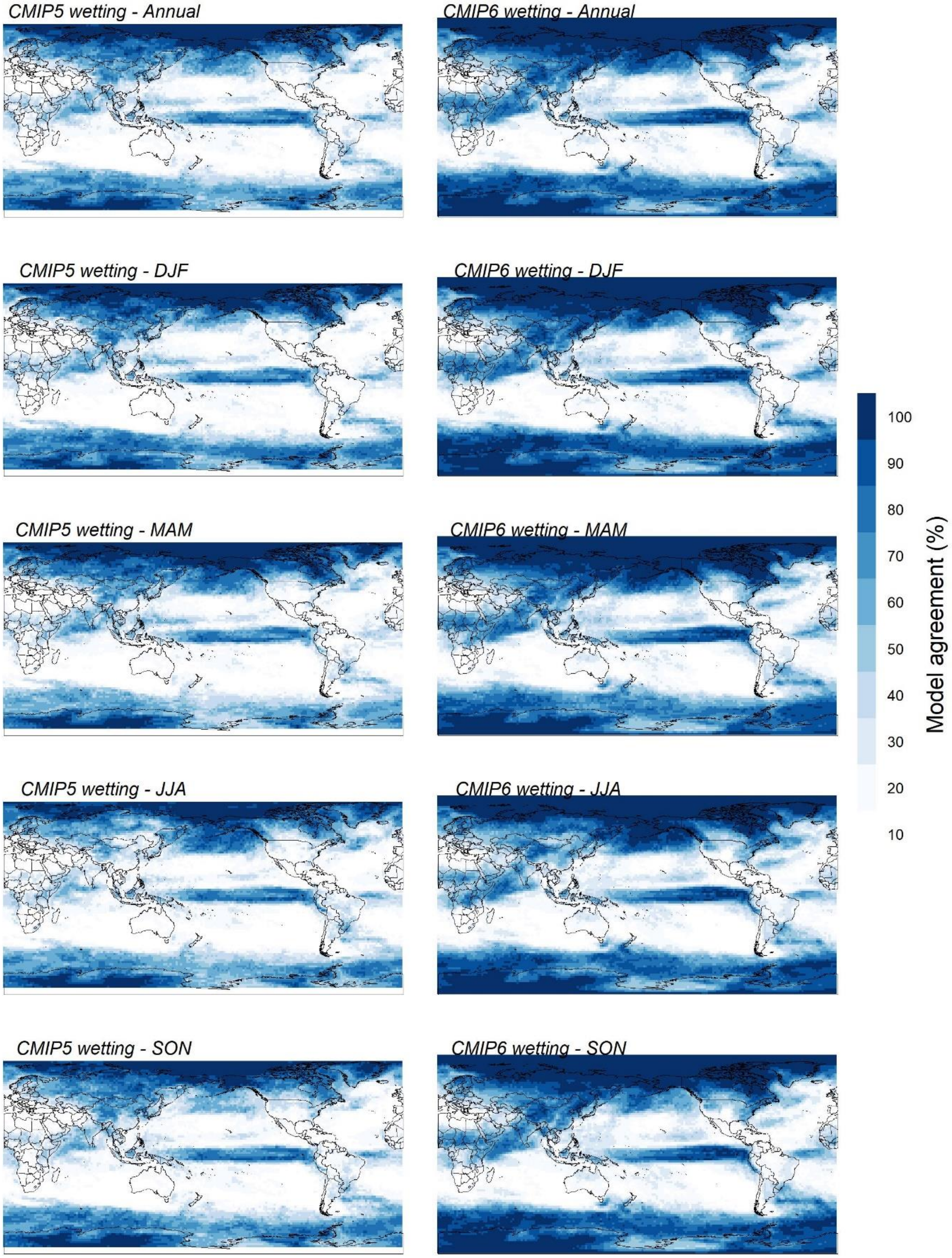


**Figure S6.** Multi-model wetting agreement for 25 CMIP5 and 42 CMIP6 models for annual and calendar seasons obtained by long-term non-parametric trends under very high emissions (RCP4.5 / SSP2-4.5).

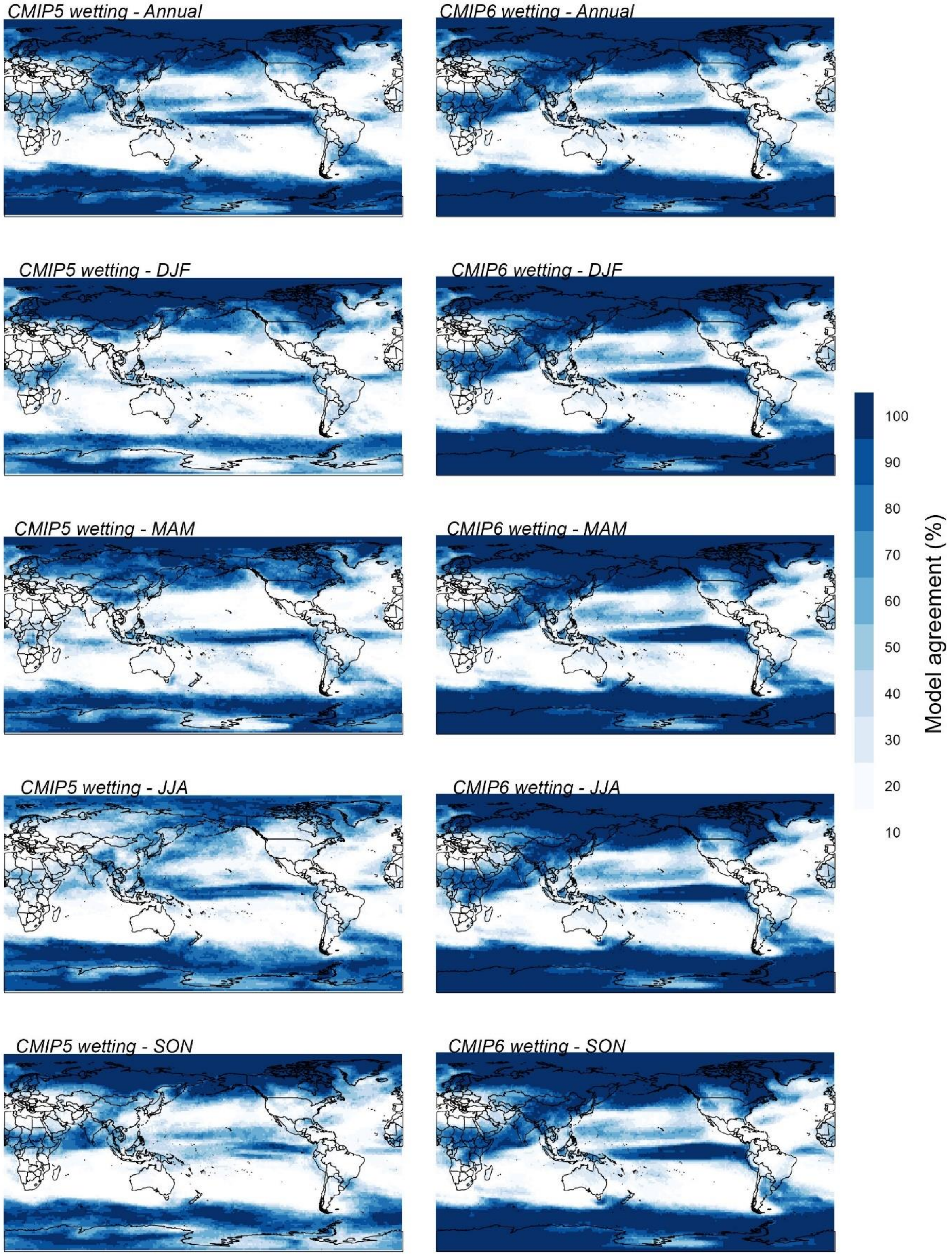


**Figure S7.** Multi-model wetting agreement for 35 CMIP5 and 44 CMIP6 models for annual and calendar seasons obtained by long-term non-parametric trends under very high emissions (RCP8.5 / SSP5-8.5).

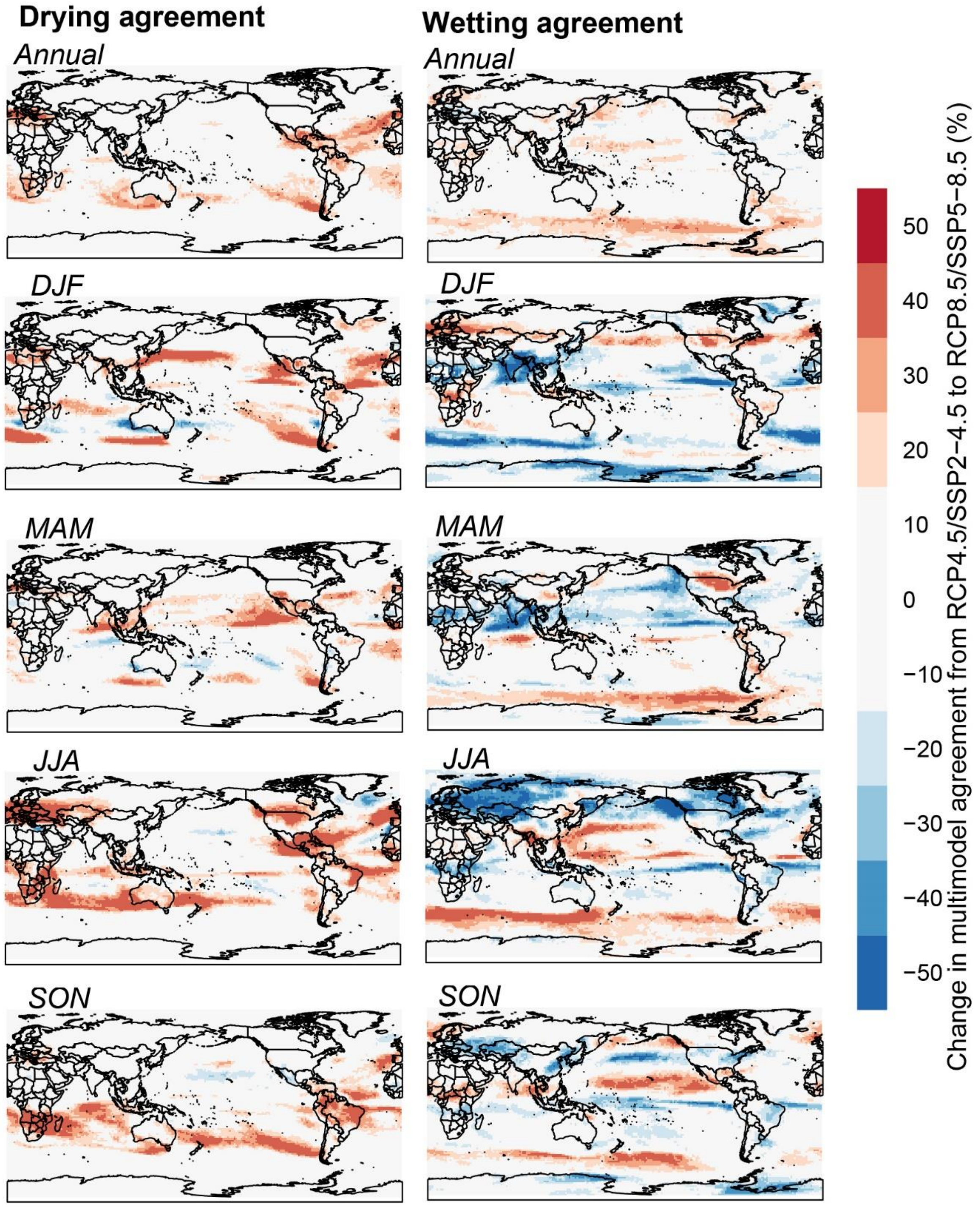


**Figure S8.** Difference in multi-model drying and wetting agreement between intermediate and very high-emissions scenarios (that is [RCP8.5 and SSP5-8.5 agreement] – [RCP4.5 and SSP2-4.5 agreement]) for CMIP5 and 6 models for annual and seasonal multi-model agreement.

**Table S1** Ensemble of 67 GCMs (25 CMIP5 and 42 CMIP6 models) forced under intermediate emissions scenarios (RCP4.5 /SSP2-4.5) used in this study.

| ID | MODEL NAME | GENERATION | INSTITUTION AND COUNTRY |
|---|---|---|---|
| 1 | ACCESS-CM2 | CMIP6 | CSIRO-ARCCSS, Australia |
| 2 | ACCESS-ESM1-5 | CMIP6 | CSIRO-BOM, Australia |
| 3 | AWI-CM-1-1-MR | CMIP6 | AWI, Germany |
| 4 | BCC-CSM2-MR | CMIP6 | BCC, CMA, China |
| 5 | CAMS-CSM1-0 | CMIP6 | CAS, China |
| 6 | CanESM5-CanOE | CMIP6 | CCCMA, Canada |
| 7 | CanESM5 | CMIP6 | CCCMA, Canada |
| 8 | CAS-ESM2-0 | CMIP6 | CAS, China |
| 9 | CESM2-WACCM | CMIP6 | NSF-DOE-NCAR, USA |
| 10 | CESM2 | CMIP6 | NSF-DOE-NCAR, USA |
| 11 | CIESM | CMIP6 | CIESM, China |
| 12 | CMCC-CM2-SR5 | CMIP6 | CMCC, Italy |
| 13 | CNRM-CM6-1-HR | CMIP6 | CMCC, Italy |
| 14 | CNRM-ESM2-1 | CMIP6 | CNRM-CERFACS, France |
| 15 | EC-Earth3-CC | CMIP6 | EC-Earth-Consortium, Europe |
| 16 | EC-Earth3-Veg-LR | CMIP6 | EC-Earth-Consortium, Europe |
| 17 | EC-Earth3-Veg | CMIP6 | EC-Earth-Consortium, Europe |
| 18 | EC-Earth3 | CMIP6 | EC-Earth-Consortium, Europe |
| 19 | FGOALS-f3-L | CMIP6 | CAS, China |
| 20 | FGOALS-g3 | CMIP6 | CAS, China |
| 21 | FIO-ESM-2-0 | CMIP6 | FIO, SOA, China |
| 22 | GFDL-CM4 | CMIP6 | NOAA, GFDL, USA |
| 23 | GFDL-ESM4 | CMIP6 | NOAA, GFDL, USA |
| 24 | GISS-E2-1-G | CMIP6 | NASA/GISS, NY, USA |
| 25 | HadGEM3-GC31-LL | CMIP6 | MOHC, UK |
| 26 | IITM-ESM | CMIP6 | CCCR, IITM, India |
| 27 | INM-CM4-8 | CMIP6 | INM, Russia |
| 28 | INM-CM5-0 | CMIP6 | INM, Russia |
| 29 | IPSL-CM6A-LR | CMIP6 | IPSL, France |
| 30 | KACE-1-0-G | CMIP6 | NIMR-KMA, Korea |
| 31 | MCM-UA-1-0 | CMIP6 | UA, AZ, USA |
| 32 | MIROC-ES2L | CMIP6 | JAMSTEC, Japan |
| 33 | MIROC6 | CMIP6 | JAMSTEC, Japan |
| 34 | MPI-ESM1-2-HR | CMIP6 | MPI-N, Germany |
| 35 | MPI-ESM1-2-LR | CMIP6 | MPI-N, Germany |
| 36 | MRI-ESM2-0 | CMIP6 | MRI, Japan |
| 37 | NESM3 | CMIP6 | NU-IST, China |
| 38 | NorESM2-LM | CMIP6 | NCC, Norway |
| 39 | NorESM2-MM | CMIP6 | NCC, Norway |
| 40 | TaiESM1 | CMIP6 | RCEC, Taiwan |
| 41 | UKESM1-0-LL | CMIP6 | MOHC-NERC, UK |
| 42 | ACCESS-CM2 | CMIP5 | CSIRO-BOM, Australia |

| | | | |
|---|---|---|---|
| 43 | bcc-csm1-1-m | CMIP5 | BCC, CMA, China |
| 44 | bcc-csm1-1 | CMIP5 | BCC, CMA, China |
| 45 | BNU-ESM | CMIP5 | BNU, China |
| 46 | CanESM2 | CMIP5 | CCCMA, Canada |
| 47 | CESM1-BGC | CMIP5 | NSF-DOE-NCAR, USA |
| 48 | CESM1-CAM5 | CMIP5 | NSF-DOE-NCAR, USA |
| 49 | CMCC-CM | CMIP5 | CMCC, Italy |
| 50 | CMCC-CMS | CMIP5 | CMCC, Italy |
| 51 | EC-EARTH | CMIP5 | EC-EARTH, Europe |
| 52 | FGOALS-g2 | CMIP5 | CAS, China |
| 53 | FGOALS-s2 | CMIP5 | CAS, China |
| 54 | FIO-ESM | CMIP5 | FIO, SOA, China |
| 55 | GISS-E2-H-CC | CMIP5 | NASA/GISS, NY, USA |
| 56 | GISS-E2-R-CC | CMIP5 | NASA/GISS, NY, USA |
| 57 | GISS-E2-R | CMIP5 | NASA/GISS, NY, USA |
| 58 | HadGEM2-AO | CMIP5 | MOHC, UK |
| 59 | HadGEM2-ES | CMIP5 | MOHC, UK |
| 60 | inmcm4 | CMIP5 | MINM, Russia |
| 61 | IPSL-CM5A-LR | CMIP5 | IPSL, France |
| 62 | IPSL-CM5A-MR | CMIP5 | IPSL, France |
| 63 | MIROC-ESM-CHEM | CMIP5 | JAMSTEC, Japan |
| 64 | MIROC-ESM | CMIP5 | JAMSTEC, Japan |
| 65 | MPI-ESM-MR | CMIP5 | MPI-N, Germany |
| 66 | MRI-CGCM3 | CMIP5 | MRI, Japan |
| 67 | NorESM1-ME | CMIP5 | NCC, Norway |

**Table S2** Ensemble of 79 GCMs (35 CMIP5 and 44 CMIP6 models) forced under very high emissions scenarios (RCP8.5 /SSP5-8.5) used in this study.

| ID | MODEL NAME | GENERATION | INSTITUTION AND COUNTRY |
|---|---|---|---|
| 1 | ACCESS1-0 | CMIP5 | CSIRO-BOM, Australia |
| 2 | ACCESS1-3 | CMIP5 | CSIRO-BOM, Australia |
| 3 | bcc-csm1-1-m | CMIP5 | BCC, CMA, China |
| 4 | bcc-csm1-1 | CMIP5 | BCC, CMA, China |
| 5 | BNU-ESM | CMIP5 | BNU, China |
| 6 | CanESM2 | CMIP5 | CCCMA, Canada |
| 7 | CCSM4 | CMIP5 | NCAR, USA |
| 8 | CESM1-BGC | CMIP5 | NSF-DOE-NCAR, USA |
| 9 | CESM1-CAM5 | CMIP5 | NSF-DOE-NCAR, USA |
| 10 | CMCC-CESM | CMIP5 | CMCC, Italy |
| 11 | CMCC-CM | CMIP5 | CMCC, Italy |
| 12 | CMCC-CMS | CMIP5 | CMCC, Italy |
| 13 | CNRM-CM5 | CMIP5 | CNRM-CERFACS, France |
| 14 | GFDL-CM3 | CMIP5 | NOAA, GFDL, USA |
| 15 | GFDL-ESM2G | CMIP5 | NOAA, GFDL, USA |
| 16 | GFDL-ESM2M | CMIP5 | NOAA, GFDL, USA |
| 17 | GISS-E2-H-CC | CMIP5 | NASA/GISS, NY, USA |
| 18 | GISS-E2-H | CMIP5 | NASA/GISS, NY, USA |
| 19 | GISS-E2-R-CC | CMIP5 | NASA/GISS, NY, USA |
| 20 | GISS-E2-R | CMIP5 | NASA/GISS, NY, USA |
| 21 | HadGEM2-AO | CMIP5 | NIMR-KMA, Korea |
| 22 | HadGEM2-CC | CMIP5 | MOHC, UK |
| 23 | INMCM4 | CMIP5 | INM, Russia |
| 24 | IPSL-CM5A-LR | CMIP5 | IPSL, France |
| 25 | IPSL-CM5A-MR | CMIP5 | IPSL, France |
| 26 | IPSL-CM5B-LR | CMIP5 | IPSL, France |
| 27 | MIROC-ESM-CHEM | CMIP5 | JAMSTEC, Japan |
| 28 | MIROC-ESM | CMIP5 | JAMSTEC, Japan |
| 29 | MIROC5 | CMIP5 | JAMSTEC, Japan |
| 30 | MPI-ESM-LR | CMIP5 | MPI-N, Germany |
| 31 | MPI-ESM-MR | CMIP5 | MPI-N, Germany |
| 32 | MRI-CGCM3 | CMIP5 | MPI-N, Germany |
| 33 | MRI-ESM1 | CMIP5 | MRI, Japan |
| 34 | NorESM1-M | CMIP5 | NCC, Norway |
| 35 | NorESM1-ME | CMIP5 | NCC, Norway |
| 36 | ACCESS-CM2 | CMIP6 | CSIRO-ARCCSS, Australia |
| 37 | ACCESS-ESM1-5 | CMIP6 | CSIRO-BOM, Australia |
| 38 | AWI-CM-1-1-MR | CMIP6 | AWI, Germany |
| 39 | BCC-CSM2-MR | CMIP6 | BCC, CMA, China |
| 40 | CAMS-CSM1-0 | CMIP6 | CAS, China |
| 41 | CanESM5-CanOE | CMIP6 | CCCMA, Canada |
| 42 | CanESM5 | CMIP6 | CCCMA, Canada |

| | | | |
|---|---|---|---|
| 43 | CAS-ESM2-0 | CMIP6 | CAS, China |
| 44 | CESM2-WACCM | CMIP6 | NSF-DOE-NCAR, USA |
| 45 | CESM2 | CMIP6 | NSF-DOE-NCAR, USA |
| 46 | CIESM | CMIP6 | CIESM, China |
| 47 | CMCC-CM2-SR5 | CMIP6 | CMCC, Italy |
| 48 | CMCC-ESM2 | CMIP6 | CMCC, Italy |
| 49 | CNRM-CM6-1-HR | CMIP6 | CNRM-CERFACS, France |
| 50 | CNRM-CM6-1 | CMIP6 | CNRM-CERFACS, France |
| 51 | CNRM-ESM2-1 | CMIP6 | CNRM-CERFACS, France |
| 52 | EC-Earth3-CC | CMIP6 | EC-Earth-Consortium, Europe |
| 53 | EC-Earth3-Veg-LR | CMIP6 | EC-Earth-Consortium, Europe |
| 54 | EC-Earth3-Veg | CMIP6 | EC-Earth-Consortium, Europe |
| 55 | EC-Earth3 | CMIP6 | EC-Earth-Consortium, Europe |
| 56 | FGOALS-f3-L | CMIP6 | CAS, China |
| 57 | FGOALS-g3 | CMIP6 | CAS, China |
| 58 | FIO-ESM-2-0 | CMIP6 | FIO, SOA, China |
| 59 | GFDL-CM4 | CMIP6 | NOAA, GFDL, USA |
| 60 | GFDL-ESM4 | CMIP6 | NOAA, GFDL, USA |
| 61 | GISS-E2-1-G | CMIP6 | NASA/GISS, NY, USA |
| 62 | GISS-E2-1-G | CMIP6 | NASA/GISS, NY, USA |
| 63 | GISS-E2-1-G | CMIP6 | NASA/GISS, NY, USA |
| 64 | HadGEM3-GC31-LL | CMIP6 | MOHC, UK |
| 65 | HadGEM3-GC31-MM | CMIP6 | MOHC, UK |
| 66 | INM-CM4-8 | CMIP6 | INM, Russia |
| 67 | INM-CM5-0 | CMIP6 | INM, Russia |
| 68 | IPSL-CM6A-LR | CMIP6 | IPSL, France |
| 69 | KACE-1-0-G | CMIP6 | NIMR-KMA, Korea |
| 70 | MIROC-ES2L | CMIP6 | JAMSTEC, Japan |
| 71 | MIROC6 | CMIP6 | JAMSTEC, Japan |
| 72 | MPI-ESM1-2-HR | CMIP6 | MPI-N, Germany |
| 73 | MPI-ESM1-2-LR | CMIP6 | MPI-N, Germany |
| 74 | MRI-ESM2-0 | CMIP6 | MRI, Japan |
| 75 | NESM3 | CMIP6 | NU-IST, China |
| 76 | NorESM2-LM | CMIP6 | NCC, Norway |
| 77 | NorESM2-MM | CMIP6 | NCC, Norway |
| 78 | TaiESM1 | CMIP6 | RCEC, Taiwan |
| 79 | UKESM1-0-LL | CMIP6 | MOHC-NERC, UK |
